# Transition-Transversion Bias Is Not Universal: A Counter Example from Grasshopper Pseudogenes

**DOI:** 10.1371/journal.pgen.0030022

**Published:** 2007-02-02

**Authors:** Irene Keller, Douda Bensasson, Richard A Nichols

**Affiliations:** 1 School of Biological and Chemical Sciences, Queen Mary, University of London, London, United Kingdom; 2 School of Biological Sciences, The University of Manchester, Manchester, United Kingdom; Princeton University, United States of America

## Abstract

Comparisons of the DNA sequences of metazoa show an excess of transitional over transversional substitutions. Part of this bias is due to the relatively high rate of mutation of methylated cytosines to thymine. Postmutation processes also introduce a bias, particularly selection for codon-usage bias in coding regions. It is generally assumed, however, that there is a universal bias in favour of transitions over transversions, possibly as a result of the underlying chemistry of mutation. Surprisingly, this underlying trend has been evaluated only in two types of metazoan, namely *Drosophila* and the Mammalia. Here, we investigate a third group, and find no such bias. We characterize the point substitution spectrum in *Podisma pedestris,* a grasshopper species with a very large genome. The accumulation of mutations was surveyed in two pseudogene families, nuclear mitochondrial and ribosomal DNA sequences. The cytosine-guanine (CpG) dinucleotides exhibit the high transition frequencies expected of methylated sites. The transition rate at other cytosine residues is significantly lower. After accounting for this methylation effect, there is no significant difference between transition and transversion rates. These results contrast with reports from other taxa and lead us to reject the hypothesis of a universal transition/transversion bias. Instead we suggest fundamental interspecific differences in point substitution processes.

## Author Summary

Some mutations occur more frequently than others. We need to understand these biases if we are to interpret the differences that have accumulated between species and individuals. Applications include estimating the time since evolutionary lineages diverged and detecting the signature of natural selection in DNA sequences. However, mutational biases have been obscured because, since mutations arose, natural selection has eliminated some whilst allowing others to persist to the present. We therefore study mutations that have accumulated in regions of the genome that are free from selection in a grasshopper with a gigantic genome. All other animal studies using this approach find an excess of mutations between DNA bases having similar biochemical properties (transitions rather than transversions). This bias has been widely interpreted as a consequence of the fundamental biochemical basis of mutation. However, once we exclude mutations associated with DNA methylation, we find no evidence of a transition bias, unlike the few comparable animal studies that make the same correction. We propose that this result indicates previously unanticipated differences between species in the selection on or mutation of their DNA.

## Introduction

While evolutionary theory assumes that mutations are random with regard to their adaptive value, it has long been recognized that this does not necessarily imply randomness in other respects. For example, some nucleotides are more mutable than others [1]. For the time being, studies of mutation inferred from genome projects [2–4] will suffer from their understandable leaning towards the study of vertebrates and species with small genomes. However, the extrapolation of the patterns observed in these studies to broad evolutionary principles has sometimes been incautious. Grasshoppers provide a valuable contrast, having gigantic genomes (the P. pedestris genome is 100 × larger than that of *Drosophila*) and associated differences in genome dynamics [5]. Indeed, they have proved to be invaluable model organisms for cytogenetic studies partly because of their massive chromosome size. In this study, we infer the mutation patterns from comparisons of DNA sequences within P. pedestris and the closely related genus *Italopodisma*.

To minimize the confounding effects of natural selection, mutation patterns have been studied in noncoding sequences such as pseudogenes [3,6–8] or “dead-on-arrival” copies of transposable elements [9]. Here, we focus on nuclear mitochondrial pseudogenes (Numts) and ribosomal DNA (rDNA) pseudogenes. Numts are ideal for the study of molecular evolution because they are abundant in many taxa, and it is straightforward to produce datasets containing many paralogous sequences [10]. In animals, Numts lose their function upon transfer into the nucleus. Freed from selective constraints, they show equal substitution rates for all three codon positions and the accumulation of stop codons and frameshift mutations [10,11].

Loss of function cannot be determined in the same way for rDNA genes, since they do not code for proteins [12]. Instead we identify as pseudogenes those sequences with substitutions in the 3′ end of the 18S coding region, which, in functional genes, is highly conserved across a wide range of taxa (indeed, in this region, there is not a single difference between *Drosophila*, various grasshoppers, and mouse). All our pseudogene sequences show multiple substitutions in this region, which strongly suggests loss of function [13]. There is convincing evidence for rDNA pseudogenes in many other species [14–20], where they could also prove useful for studies on molecular evolution.

Both Numts and rDNA pseudogenes are distributed widely throughout these genomes and are, consequently, expected to reflect genome-wide substitution patterns. The evidence for their distribution comes from fluorescent in situ hybridisation: in the case of Numts the whole chromosome complement of *Italopodisma* was labelled by a mitochondrial cytochrome oxidase I gene probe suggesting that copies are distributed quite uniformly over all chromosomes [21]. The rDNA sequences are also widespread but more aggregated; they can be detected in up to seven different loci on different chromosomes where they seem to be tandemly arrayed (P. Veltsos, personal communication). Past work shows that active rDNA sequences map to the GC rich heterochromatin of these grasshoppers [22].

Here we quantify the neutral point substitution spectrum in these two different categories of sequence and demonstrate that substitutional bias for transitions over transversions is not a universal rule.

## Results

### Assessment of Data Quality

Our analysis attributed 301 of the point substitutions seen in Numts to mutations that arose in the nucleus. Some of the Numt sequences were obtained by a protocol that selected against the most recently arising Numts (see below). There was no detectable difference between the substitution spectrum in this sample of old pseudogenes and the randomly selected sequences (likelihood ratio test, *χ* ^2^ = 2.06, *df* = 5, *p* = 0.91).

The efficacy of the procedure used to distinguish substitutions that had occurred in the nuclear DNA from those that had taken place in mitochondria was confirmed by the absence of a significant codon-position bias for the unique substitutions (*χ* ^2^ = 4.43, *df* = 2, *p* = 0.11). The number at the third position was actually slightly lower than the expected value (81 versus 96.9, with a correction for available sites). Conversely, as expected for a protein-coding gene evolving under selective constraints, the substitutions attributed to the genuine mitochondrial lineage showed a highly significant codon position bias (*χ* ^2^ = 12.72, *df* = 2, *p* = 0.0017), in which mutations at the third codon position were overrepresented (94 versus 74.5). As expected in pseudogenes, complementary mutations occurred at similar frequencies in the Numt data (*G*-test, *G* = 11.47, *df* = 6, *p* = 0.07).

There were 154 point mutations that could be analysed in the rDNA pseudogenes. Of these, 31 were observed in the section paralogous to the 18S coding region and 123 in the section paralogous to the internal transcribed spacer 1. As expected for point substitutions accumulating in nonfunctional DNA, the proportion of mutating positions is similar in the two DNA regions (test for equality of proportions, *χ* ^2^ = 1.66, *df* = 1, *p* = 0.20). Because rDNA is not protein coding we cannot assess the codon positions of the substitutions. Complementary mutations occurred at similar frequencies (*G*-test, *G* = 3.44, *df* = 6, *p* = 0.75).

**Figure 1:**
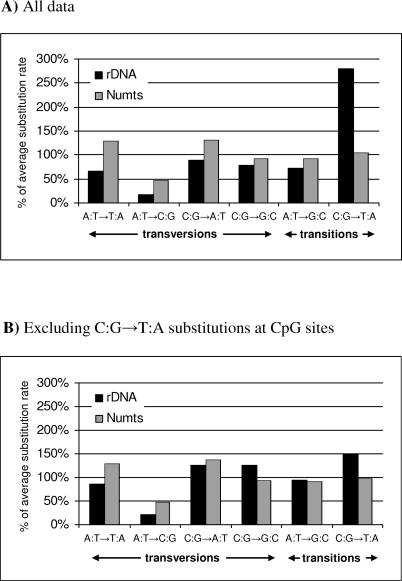
Neutral Point Substitution Patterns in Two Types of Grasshopper Pseudogenes (A and B) Frequency of the different substitutions in rDNA pseudogenes (black) and Numts (grey) expressed as a percentage of the average substitution rate.

The substitution rates observed in the two datasets are visualized in Figure 1. We found no significant difference in the spectrum of point substitutions between Numts and rDNA pseudogenes once we account for differences in the number of sites available for different types of substitution (glm, *χ* ^2^ = 10.42, *df* = 11, *p* = 0.49).

### No Transition Bias after Accounting for Hypermutability of Cytosines Adjacent to Guanines

Analysis of deviance revealed two significant biases in the point substitution pattern in *Podisma*. Firstly, the rate of C:G → T:A transitions was significantly elevated at cytosine residues adjacent to guanines (CpG sites) (glm, *χ* ^2^ = 74.75, *df* = 1, *p* < 0.001). This effect of CpG hypermutability is illustrated in Figure 1 where the frequency of the different substitutions is shown before (Figure 1A) and after (Figure 1B) the exclusion of CpG sites. There is no discernable effect of excluding CpG sites on the transition bias observed in Numts, simply because of the rarity of CpG sites in this pseudogene family (4 CpGs in 849 bp). Although there is significant heterogeneity of substitution rates after excluding CpG sites (glm, *χ* ^2^ = 48.68, *df* = 5, *p* < 0.001), there is no significant transition bias (glm, *χ* ^2^ = 0.45, *df* = 1, *p* = 0.504).

The significant heterogeneity in the point substitution spectrum shows that although we do not detect transition bias, there is sufficient data to identify effects other than CpG hypermutability. More specifically, we observed a significant lack of A:T → C:G transversions relative to all other substitutions (glm, *χ* ^2^ = 42.31, *df* = 1, *p* < 0.001).

In our model, we corrected for differences in the number of sites available for different types of point substitution. Even without this correction (which is more important in comparisons that differ in the base composition of the nucleotides involved), we observed 126 transitions and 267 transversions after excluding transitions that could have occurred at methylated cytosines. This ratio (1:2.1) was not significantly different from the 1:2 ratio expected from equal rates of transition and transversion (binomial exact test: *p =* 0.63), but was clearly different from the ratio of 2:1 (binomial exact test: *p* < 2 × 10^−16^), which was observed in human pseudogenes after the exclusion of transitions at CpG sites [3].

Could multiple independent substitutions occurring at individual sites lead to an underestimation of the transition bias? We address this possibility using a maximum likelihood approach, which accounts for such multiple hits (see Material and Methods). This showed that, after the exclusion of methylation effects, the transition rate was 1.13 times the transversion rate, which is not significantly different from even (95% confidence interval = 0.89 − 1.42). Given that there are twice as many possible transversions an even rate is sometimes reported as 1:2, which corresponds to 1:1.77 in our case.

### Mutational Bias towards Adenine and Thymine

**Figure 2:**
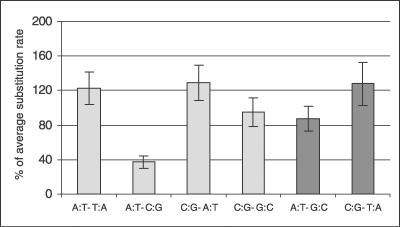
Neutral Point Substitution Pattern in Grasshopper Pseudogenes Accounting for Multiple Substitutions Maximum likelihood estimates of the frequency (with associated standard errors) of the different substitutions in grasshopper pseudogenes expressed as a percentage of the average substitution rate (C:G → T:A substitutions at CpG sites are excluded) (light grey, transversions; dark grey, transitions).

Whether or not CpG sites are included, the pattern of point substitutions results in an overall bias in favour of cytosine and guanine changing to adenine and thymine (previous section and Figures 1 and 2). However, these differences are only significant for the A:T → C:G transversion, which occurs approximately three times less frequently than the other five substitutions (likelihood ratio test, *χ* ^2^ = 48.7, *df* = 1, *p* < 0.001).

As a consequence, the equilibrium base composition is biased towards A and T (A + T), with a predicted A + T content of 58% for Numts and 60% for rDNA pseudogenes. In both datasets, the unequal numbers of A + T → G + C and G + C → A + T changes suggest that the base composition is not presently at equilibrium (rDNA pseudogenes: 16 A + T → G + C and 35 G + C → A + T, *p* = 0.008; Numts: 118 A + T → G + C and 49 G + C → A + T, *p* < 0.001). The A + T content of rDNA pseudogenes is increasing, while the opposite is the case for the currently very A + T rich Numts, and both are expected to equilibrate at a similar final AT content (∼60%).

### Comparison between *Podisma* and *Drosophila*

**Figure 3:**
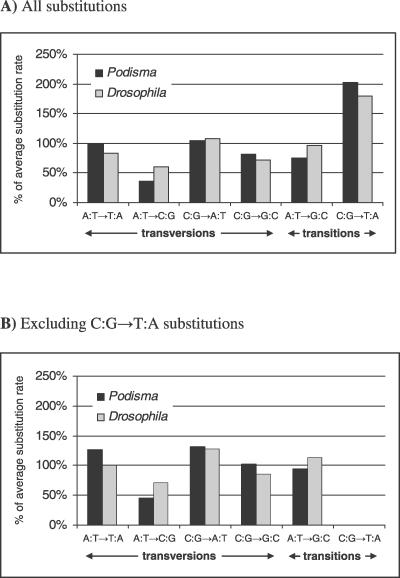
Neutral Point Substitution Patterns in the Grasshopper *Podisma* and the Fruit Fly *Drosophila* Frequency of the different point substitutions in *Podisma* (black) and *Drosophila* (grey) (data from Petrov and Hartl [9]) expressed as a percentage of the average substitution rate.

The point substitution spectra observed in the two species are illustrated in Figure 3. An analysis of deviance showed that the substitution patterns did not differ significantly between the two species (glm, *F*_5,6_ = 0.41, *p* = 0.83). If all C:G → T:A transitions were excluded, as in Petrov and Hartl [9], to allow comparison of *Drosophila* with methylated genomes, the point substitution spectra of the two species showed a small but significant difference (glm, χ^2^ = 10.89, *df* = 4, *p* = 0.03).

## Discussion

### Equal Transition:Transversion Ratio at Non-CpG Sites

It is generally assumed that the ratio of transitions (ts) to transversions (tv) is higher in animal nuclear genomes than the 1:2 ratio expected if all substitutions were equally likely, while the relative transition rate is even higher in their mitochondrial DNA [1,23]. As a consequence, some of the best known models of sequence evolution assume that the most relevant difference in substitution rate occurs between transitions and transversions and, consequently, allow for different rates for these two categories (e.g., [24,25]). In many species, vertebrates in particular, this rule appears to hold quite well.

Part of the higher rate of transitions in vertebrates can be attributed to the effect of methylation. At vertebrate CpG sites methylation is widespread [26], and the deamination of methylated cytosines leads to highly elevated transition rates at these sites [2–4,6–8,27,28]. There is an additional underlying effect. Transitions are more frequent than transversions even after accounting for the hypermutability of CpG sites [1,3,6,27].

In *Podisma*, however, this is clearly not the case. The Orthoptera also exhibit CpG methylation [29], and *Podisma* has a correspondingly elevated frequency of C:G → T:A transitions at CpG dinucleotides. However, after the exclusion of CpG sites, we observe no underlying effect equivalent to that in vertebrates. The ts:tv ratio is not significantly different from 1:2, as would be expected under a uniform mutation rate (because there are twice as many possible transversions). The only comparable insect dataset, from *Drosophila* [9], shows a significantly different pattern, with a ts:tv ratio of 1:1.22. Although the transition frequency in *Drosophila* is lower than vertebrate estimates, the ts:tv ratio deviates significantly from the expectation under a uniform mutation rate (*χ* ^2^-test, *χ* ^2^ = 38.35, *df* = 1, *p* < 0.001). This increased transition rate is mainly attributable to C:G → T:A substitutions, but it cannot be due to CpG hypermutability because few CpG sites are methylated in the *Drosophila* genome [30]. It has, however, proved difficult to characterise DNA methylation in insects [31], and the assumption that DNA methylation is completely absent from the *Drosophila* genome has been challenged by recent studies [30,32]. In contrast to other species, most methylated cytosines are found in the non-CpG dinucleotides of *Drosophila*. Interestingly, retrotransposable elements, the very type of sequence analysed by Petrov and Hartl [9], have been identified as potential targets for DNA methylation [33]. It is an exciting if, at present, highly speculative possibility that after the exclusion of methylation effects, *Drosophila* might also no longer exhibit a transition bias.

### Evolution of Base Composition

In both pseudogene families, the rate of substitutions from C + G to A + T is higher than the rate of changes in the opposite direction (Figures 1 and 2), leading to a predicted equilibrium A + T content of 58% or 60%. At present, it is unclear why the A:T → C:G transversion occurs at a particularly low frequency. The predicted base composition matches empirical measures of the actual genomic base composition of a variety of grasshopper species, which all have between 51% and 60% A + T content [34].

Mutational biases for C + G → A + T substitutions seem to be very common and are reported in bacteria, insects, and mammals [6–9,35–40]. Results from *Drosophila* [41] and mammals [42], suggest patterns of point substitution that have recently changed in these groups. In these grasshoppers, however, we observe no evidence for a change in the pattern of nucleotide substitution when comparing a random sample of Numts to one that is enriched for more ancient Numts (*G*-test, *G* = 8.45, *df* = 11, *p* = 0.67).

Insertion into a different position in the genome and loss of function seem the most likely explanations for the nonequilibrium base composition observed in our sequences. Generally, newly inserted sequences tend to evolve towards a G + C content that is similar to that of the surrounding DNA [36]. Consequently, the Numts (78% A + T), which arose recently from mtDNA, reflect the high A + T content typical of insect mitochondrial DNA [43,44], but are evolving towards a base composition more characteristic of the nuclear genome. The opposite trend is observed for the rDNA pseudogenes whose current A + T content of 39% is expected to increase to an equilibrium value of 58%. This suggests that the G + C content of functional rDNA sequences might be maintained at above-equilibrium values by selection. This interpretation is consistent with the observation that genes generally tend to be G + C rich [45].

The point substitution spectra inferred from the two pseudogene families are similar in spite of differences in their base composition, genomic location, and possibly mode of molecular evolution. Similarly, results from *Drosophila* showed no differences in the substitution and indel spectra inferred from single-copy [46] and multi-copy sequences [46,47], and although there is heterogeneity in the G + C content of the D. melanogaster genome, the pattern of point substitution does not differ in regions with different base composition [48]. The case is quite different in mammals that show patterns of point substitution that differ among genomic regions [36,42,49].

### Conclusions

The point substitution spectrum in *Podisma* is similar in some respects to what has been observed in other species (e.g., hypermutability of CpG sites and mutational bias towards A + T). A remarkable difference, however, is the complete absence of a transition bias.

The transition bias observed in other metazoans could be caused by a mutational bias due to intrinsic properties of DNA. Alternatively, in coding regions, the bias could be explained by selection on nonsynonymous transversions. Both transitions and transversions can change the amino acid composition of the corresponding protein, but the biochemical difference in the protein product tends to be greater for transversions [50]. Consequently, there is likely to be greater purifying selection against transversions. Selection could therefore favour DNA repair systems that are particularly efficient at preventing transversions, which in turn would affect the observed substitution patterns across the whole genome including noncoding regions. The intensity of purifying selection may vary between genes for many reasons, including differing constraints on protein structure and the importance of codon usage bias. For example, in *Plasmodium* genes the ts:tv ratio varies enormously from 1:0.07 to 1:3.54 [51].

The efficacy of selection is also likely to differ between species, as it may be affected by effective population size [52] or the recombination rate [53], which in turn could explain interspecific differences in the ts:tv ratio. The exceptionally large genome size of P. pedestris [54] might indeed indicate a reduced efficacy of selection in this species, possibly as a consequence of a small effective population size [55]. However, it seems unlikely that transition bias in other species is explained exclusively by the action of selection on individual mutations. In that case, selection on individual transitions in human pseudogenes would need to explain the transition bias in humans [3], which also have a large genome size and small effective population size [55].

Thus far, the evidence suggests that a mutational bias explains transition bias, at least in Caenorhabditis elegans, even in the absence of selection acting on individual mutations [56]. Differences in point substitutional spectra, and even in the ratios of transitions to transversions, have been detected as a result of genetic disorders in humans [57]. It is therefore plausible that the underlying chemistry of mutation and DNA repair will affect the transition bias and could differ in *Podisma*.

The results of this study emphasise the importance of evaluating neutral point substitution spectra across a wide range of taxa. Such a broad taxonomic approach will not only reveal potential differences between groups, but also hopefully provide clues as to what mechanistic or selective forces might be responsible for them.

## Materials and Methods

### Numt sequencing.

All Numt sequences were obtained from P. pedestris (Orthoptera, Acrididae) and the closely related Italopodisma sp. and are paralogous to 643 bp of the mitochondrial ND5 region. Classification within the genus *Italopodisma* is difficult as it is based exclusively on subtle differences in the male genitalia [58]. For this reason, we do not distinguish between different *Italopodisma* species. We use the genus name *Podisma* when we refer to both P. pedestris and Italopodisma sp.

The molecular methods used to generate these data have been described in detail elsewhere [59]. Briefly, PCR primers were designed based on the ND5 sequence from the grasshopper Locusta migratoria. These primers amplify mtDNA sequences as well as paralogous ND5-like Numts in all studied species. The PCR products were cloned, reamplified by PCR from individual colonies, and sequenced from both strands. More than 80% of the Numt sequences were obtained from PCR products amplified using a high-fidelity polymerase (Pfu polymerase). The low error rate of this enzyme had been confirmed in preliminary tests [5].

In some cases, we enriched for Numts of a particular evolutionary age by digesting total genomic DNA with a restriction enzyme prior to PCR. More specifically, restriction enzymes were chosen so that their recognition sequences would be specific to mtDNA and recently arising Numt sequences. Digestion of total genomic DNA with such enzymes ensures that mtDNA and recently arising pseudogenes will not be amplified, cloned, or selected for further study. Phylogenetic analysis shows that we targeted and successfully obtained 23 anciently arising Numt sequences or Numts of an intermediate age relative to most other Numts. These pseudogenes do, however, fall within the same clade as some of the randomly sampled Numts. Together with the data from Bensasson et al. [59], this gave a total of 57 P. pedestris sequences and 34 Italopodisma sp. sequences.

To determine which ND5 sequences were functional and which were pseudogenes, DNA was extracted using a protocol that enriches for mtDNA [59]. PCR amplification from each of these templates predominantly produced one type of sequence, which was assumed to be the mtDNA sequence. Consistent with our designation of mitochondrial sequences, most of the Numts studied contained frameshift mutations (see [59] for fuller discussion).

### Separation of nuclear and mitochondrial changes.

As we are interested in the pattern of point substitutions in noncoding DNA, it is essential to exclude from our analysis mutations that arose in the mitochondria while the sequence was still under selective constraints. It has been shown that an effective separation of nuclear and mitochondrial substitutions can be achieved by counting only unique substitutions as nuclear [5,35]. This is based on the reasoning that, in relatively undiverged sequences, nucleotide differences that are shared by two or more Numts are more likely to be the result of common ancestry than of multiple independent mutations. Since many Numts were derived independently from divergent mitochondrial ancestors [59], many of these shared differences probably arose in the mitochondria. This method for distinguishing nuclear and mitochondrial mutations is similar in effect to a maximum parsimony approach (e.g., [9]). The only difference between the two methods is that maximum parsimony can, in some cases, ascribe shared substitutions resulting from multiple hits to nuclear evolution, while the approach based on unique substitutions will always count them as mitochondrial.

Divergence analysis had shown that some Numts were more similar to mtDNA sequences from another species, Parapodisma mikado, than to the actual P. pedestris or Italopodisma sp. mtDNA sequences (unpublished data). To reduce the chance that nucleotide sites representing such an ancestral mitochondrial state were counted as unique substitutions, a P. mikado was included in the alignments.

### The rDNA pseudogene sequencing.

All pseudogenes used in this section are paralogous to 494 bp of the rDNA genic region and correspond to the 3′ end of the 18S (119bp) and the entire internal transcribed spacer 1. The rDNA was PCR amplified using the primers 18S(f) and ITS6(r) [60] and the standard PCR protocol described in Keller et al. [13]. A total of seven of the sequences were obtained from RNA templates using RT-PCR (see [13]). The PCR products were cloned, the inserts reamplified from single colonies, and sequenced from both directions. A total of 35 pseudogene sequences were included in the analysis.

### The treatment of rDNA sequences.

The rDNA sequences differ from the Numts, which are known to be nonfunctional in the nucleus. However, the rDNA mutations have been identified by comparison between sequences within clades of pseudogenes [13] and so appear almost certain to have occurred in nonfunctional genes. This interpretation is supported by comparison of mutation rates in the internal transcribed spacer 1 and the 18S gene, which evolve at highly different rates in functional copies [61], but which show no significant difference in our comparisons (test for equality of proportions, χ^2^ = 1.66, *df* = 1, *p* = 0.20).

The inclusion of sequence differences caused by PCR errors could seriously distort our rDNA results, because they are biased in favour of particular changes [62]. We therefore identified mutations that occurred in vivo based on the criterion that they occurred in more than one sequence in the alignment. Each of these nonunique differences was counted as a single mutation. The multiple occurrences could be explained either because the sequences shared a difference from the consensus by simple inheritance from a common ancestor, or because mutations had spread horizontally through the rDNA multigene family by the processes causing concerted evolution (see [1]). Our approach is highly conservative, but will not bias the results unless concerted evolution favours particular substitutions. We expect any such bias to be detected by comparison of the rDNA and Numt results.

### Pattern of nucleotide substitutions.

We used BioEdit 7.0.1 [63] to create separate alignments of the Numt sequences from *P. pedestris,* the Numt sequences from Italopodisma sp., and the rDNA pseudogene sequences from P. pedestris. All three alignments were verified by eye. To allow for the automated detection of appropriate rDNA mutations (see above), the alignment was edited by hand to replace single differences, and all but one of the multiple differences, with the ancestral base. Unique substitutions were then identified using the perl script unique.pl written by D. Bensasson. The identification of unique substitutions and the two bases immediately adjacent to the mutating nucleotide (and therefore CpG sites) was automated using unique.pl. For the Numt data, unique.pl also outputs the codon position of all unique and nonunique substitutions. If more than one ancestral state was possible for a given substitution, the most likely ancestral nucleotide from a phylogenetic tree of all sequences was called manually. All ambiguous cases were excluded from further analysis.

The substitution rates were normalized to account for unequal frequencies of the four bases (*i* = A, G, C, or T). More precisely, if *N*_i → j_ is the number of times a nucleotide of type *i* has mutated to a nucleotide of type *j,* and *T*_i → j_ is the total number of type *i* nucleotides in which a change from *i* to *j* could have been observed, then the different substitution rates are defined as *S*_i → j_ = (*N*_i → j_)/(*T*_i → j_). *T*_i → j_ was estimated using unique.pl from the total number of type *i* nucleotides corrected for the fact that, in the Numt data, our unique approach resulted in some positions being unavailable for some substitutions. For example, it will not be possible to observe a unique T → G change at a position where most sequences have a T but some have a G. To represent complementary mutations, we use the following notation: A:T → G:C represents A → G and T → C. The notation A + T → G + C, on the other hand, summarizes all four possible changes that lead from A or T to G or C.

The equilibrium G + C content, f*, was estimated as f* = *v*/(*u* + *v*), where *v* was the rate (per nucleotide) of A + T → G + C mutations and *u* the rate of G + C → A + T changes [64]. The mutation rate *u* was estimated after the exclusion of substitutions at CpG sites.

### Statistical analyses.

All statistical tests were carried out using “R” 2.1.0 [65]. A generalised linear model (analysis of deviance) with Poisson errors was fitted to the data. This model included three factors: type of pseudogene, type of substitution, and methylation sensitivity, and their interaction effects on the number of observed changes. The number of bases available for a given substitution was included in the models as an offset [66]. Terms were dropped from the model if their deletion did not cause a significant increase in deviance. As expected for pseudogenes in nuclear genomes, there were no significant strand asymmetries in the substitution biases we observe (see Results) and so “type of substitution” was treated as a factor with six levels (Figures 1–3). The final model was checked for overdispersion of residual deviance. For “type of substitution,” factor levels were combined following the general recommendations of Crawley [66], again, provided this did not lead to a significant increase in deviance.

### Maximum likelihood estimation accounting for the possibility of multiple hits.

The maximum likelihood estimates of the mutation rates were calculated using the following expression for the likelihood:

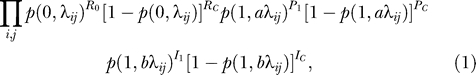
where *p*(0, λ*_ij_*) is the Poisson probability density of 0 mutations producing base *j* at a site where the ancestral base was *i,* given a mutation rate λ*_ij_* (mutations per site over the whole genealogy). The first term of the product corresponds to the rDNA data. The parameter *R*_0_ is the number of sites at which there were no mutations from *i* to *j* scored over the whole rDNA genealogy (the category that can be unambiguously identified), and *R*_C_ is the number of sites in other categories. The second term corresponds to the Numt data from *Podisma*. The parameter *P*_1_ is the number of sites where there has been a single mutation from *i* to *j* over the whole genealogy (the category that can be unambiguously identified for Numt data), and *P*_C_ is the number of sites in other categories. The appropriate Poisson density is determined by the rate *a*λ*_ij_,* in which the parameter *a* allows for the different total length of the Numt and rDNA genealogies. The final term in the product is of the same form, but for the *Italopodisma* Numt data. Notice that we can detect, and allow for, multiple mutations at the same site if they occur in different branches of the same genealogy. We have neglected multiple mutations in the same branch, but the most common of these would be in the *Podisma* Numt data, and we calculate the probability of a double mutation in the same branch of the genealogy (with 57 terminal branches) to be *p*(1, *a*λ*_ij_*)^2^/57, which is 256 times smaller than the one-mutation probability in the largest case.

The maximum likelihood values of the parameters, standard errors, and confidence intervals were determined using the “mle” function in the statistical package R [65]. The significance of differences between the mutation rates were established by comparing the deviance (twice the log likelihood ratio) of models with different rates (λ*_ij_*) to models in which some rates were constrained to be identical.

### Comparison to *Drosophila.*

The point substitution spectrum in *Podisma* was compared to that inferred from transposable elements in the *Drosophila* genome using analysis of deviance as detailed above. The proportion of substitutions from Figure 3 in Petrov and Hartl [9] was converted back into actual counts using the base composition of the relevant *Drosophila* sequences. We compensated for overdispersion by refitting the model with quasipoisson errors and using *F*-tests rather than χ^2^-test for model comparison [67].

Petrov and Hartl [9] examine the substitution spectrum in transposable elements and not in pseudogenes, because of the paucity of pseudogenes in the *Drosophila* genome. They show that 5′ truncated copies of non-LTR retrotransposable elements are inactive and essentially evolve as pseudogenes [9]. There are very few Numt sequences in *Drosophila,* only three have been identified in the sequenced D. melanogaster genome [10], but the longest of these (566 bp) has been studied in detail [47]. No major differences were detected in the patterns of molecular evolution of this Numt, mobile elements, and the few other types of pseudogenes for which sequence was available. We therefore expect that the substitution spectrum reported by Petrov and Hartl [9] is comparable to that observed in other nonfunctional sequences such as pseudogenes.

## Supporting Information

### Accession numbers

The EMBL database (http://www.ebi.ac.uk/embl) sequences used in this article under the following accession numbers are: for *P. pedestris* and Italopodisma sp. mitochondrial DNA (AF085501–AF085505) and Numts (AF085508–AF085524, AF085526–AF085538, AF085575–AF085578, AF085539–AF085545, AF085547–AF085550, AF085552–AF085574, EF088292–EF088294, EF088296–EF088309, EF088313, and EF088319–EF088323); for rDNA pseudogenes (AM183587, AM183588, AM183591–AM183594, AM183596–AM183608, AM183610–AM183613, AM183616–AM183624, and AM238436–AM238438); for *Parapodisma mikado* (AF085506)*;* for *Locusta migratoria* (X80245); and for Drosophila sp. (AF012030–AF012035, AF012037–AF012052, U62715–U62731, U65653).

## Abbreviations

CpG: cytosine residue adjacent to guanine in a DNA sequence
Numt(s): nuclear copy of mitochondrial DNA sequence(s)
rDNA: ribosomal DNA
ts: transition
tv: transversion
